# Multiparametric MRI Evaluation of Oropharyngeal Squamous Cell Carcinoma. A Mono-Institutional Study

**DOI:** 10.3390/jcm10173865

**Published:** 2021-08-28

**Authors:** Francesca Piludu, Simona Marzi, Emma Gangemi, Alessia Farneti, Laura Marucci, Aldo Venuti, Maria Benevolo, Barbara Pichi, Raul Pellini, Francesca Sperati, Renato Covello, Giuseppe Sanguineti, Antonello Vidiri

**Affiliations:** 1 Department of Radiology and Diagnostic Imaging, IRCCS Regina Elena National Cancer Institute, Via Elio Chianesi 53, 00144 Rome, Italy; francesca.piludu@ifo.gov.it (F.P.); emma_gan86@yhaoo.it (E.G.); 2 Medical Physics Laboratory, IRCCS Regina Elena National Cancer Institute, Via Elio Chianesi 53, 00144 Rome, Italy; simona.marzi@ifo.gov.it; 3 Center for Integrated Research, Departmental Faculty of Medicine and Surgery, University Campus Bio-Medico of Rome, Via Álvaro del Portillo, 33, 00128 Rome, Italy; 4 Department of Radiotherapy, IRCCS Regina Elena National Cancer Institute, Via Elio Chianesi 53, 00144 Rome, Italy; alessia.farneti@ifo.gov.it (A.F.); laura.marucci@ifo.gov.it (L.M.); giuseppe.sanguineti@ifo.gov.it (G.S.); 5 HPV Unit (UOSD), Department of Tumor Immunology and Immunotherapy, IRCCS Regina Elena National Cancer Institute, Via Elio Chianesi 53, 00144 Rome, Italy; aldo.venuti@ifo.gov.it; 6 Department of Pathology, IRCCS Regina Elena National Cancer Institute, Via Elio Chianesi 53, 00144 Rome, Italy; maria.benevolo@ifo.gov.it (M.B.); renato.covello@ifo.gov.it (R.C.); 7 Department of Otolaryngology and Head and Neck Surgery, Regina Elena National Cancer Institute, Via Elio Chianesi 53, 00144 Rome, Italy; barbara.pichi@ifo.gov.it (B.P.); raul.pellini@ifo.gov.it (R.P.); 8 Biostatistics-Scientific Direction, IRCCS San Gallicano Dermatological Institute, Via Elio Chianesi 53, 00144 Rome, Italy; francesca.sperati@ifo.gov.it

**Keywords:** magnetic resonance imaging, diffusion magnetic resonance imaging, perfusion magnetic resonance imaging, oropharyngeal squamous cell carcinoma, human papillomavirus

## Abstract

The aim of this paper is to define the pre-treatment radiological characteristics of oropharyngeal squamous cell carcinoma (OPSCC) using morphological and non-morphological magnetic resonance imaging (MRI), based on HPV status, in a single-institution cohort. In total, 100 patients affected by OPSCC were prospectively enrolled in the present study. All patients underwent 1.5T MR with standard sequences, including diffusion-weighted imaging with and intravoxel incoherent motion (IVIM-DWI) technique and a dynamic contrast-enhanced (DCE) MRI. For all patients, human papillomavirus (HPV) status was available. No statistically significant differences in the volume of primary tumors (PTs) and lymph nodes (LNs) were observed based on HPV status. When comparing the two patient groups, no significant differences were found for the PT radiologic characteristics (presence of well-defined borders, exophytic growth, ulceration, and necrosis) and LN morphology (solid/cystic/necrotic). Tumor subsite, smoking status, and alcohol intake significantly differed based on HPV status, as well as ADC and D_t_ values of both PTs and LNs. We detected no significant difference in DCE-MRI parameters by HPV status. Based on a multivariate logistic regression model, the combination of clinical factors, such as tumor subsite and alcohol habits, with the perfusion-free diffusion coefficient D_t_ of LNs, may help to accurately discriminate OPSCC by HPV status.

## 1. Introduction

Squamous cell carcinoma (SCC) is a common malignant tumor affecting the skin. A peculiar feature of this tumor is the abnormal and quick growth of keratinocytes in the epidermis, often secondary to ultraviolet or sunlight exposure [1]. When this tumor involves the head and neck area, it may be particularly aggressive and can account for up to 90% of all oral malignancies [2]. In recent decades, there has been a dramatically increased incidence in oropharyngeal squamous cell carcinoma (OPSCC) associated with human papillomavirus (HPV) infection [3].

At present, HPV-related OPSCC has been found to be a separate subtype of head and neck squamous cell carcinoma (HNSCC), characterized by peculiar biologic features, clinical course, and response to treatment [4]. HPV-OPSCC has been shown to have a better prognosis [5]. A meta-analysis of retrospective studies by Ragin et al. [6] indicated that patients with HPV-positive OPSCC exhibited markedly reduced risk of mortality compared to patients affected by HPV-negative OPSCC. Fine needle aspiration cytology (FNAC) with ultrasonography, computed tomography (CT), and MRI are the techniques generally used for the local staging of OPSCC, while positron emission tomography (PET)-CT is useful in locally advanced stages and for carcinomas of unknown primary origin [7,8,9,10,11]. Several studies [11,12,13,14] have reported differences in imaging features of OPSCC by HPV status. HPV-related lymph nodes (LNs) often tend to present with large cystic masses, with a clinically and radiologically smaller or even occult primary tumor (PT) [13]. In recent years, there has been an increasing focus on evaluating OPSCC with a multiparametric MRI approach using non-morphological imaging, i.e., diffusion-weighted imaging (DWI) and perfusion-weighed dynamic contrast-enhanced MRI [15,16,17]. DWI allows for the gathering of information about cellularity, and the apparent diffusion coefficient (ADC) has been found to correlate with cell density and proliferation [16]. DCE provides information about tissue vascularity and is also related to various histopathological features, such as vessel count, total vessel area, and microvessel density [17,18]. The possibility of predicting HPV status by imaging biomarkers can be used as a non-invasive and cost-effective tool in patients who have undergone imaging for staging.

In this study, we analyze the pre-treatment MR characteristics of OPSCC in a single-institution patient cohort. In particular, we focus on morphological and non-morphological MRI features of both primary tumors and neck nodes, including diffusion-weighted and perfusion-weighted sequences, after separating patients by HPV status.

## 2. Materials and Methods

### 2.1. Patients

The study protocol was authorized by the local Institutional Review Board (approval number RS716/15), and specific, informed consent was obtained from each patient. The criteria for inclusion were: (i) age older than 18 years; (ii) OPSCC confirmed by biopsy; and (iii) availability of HPV status. Exclusion criteria included: (i) any contraindication to MR examination; (ii) prior surgery as excisional biopsy of the tumor or neck nodes; (iii) prior chemotherapy (CHT), including induction CHT or radiotherapy to the primary disease and the neck; (iv) unknown HPV status; and (v) distant metastases at presentation (M1). The disease was staged following the 8th edition of the AJCC (American Joint Committee on Cancer) Cancer Staging Manual for HNSCC [19].

### 2.2. HPV Detection

Two techniques (p16 immunohistochemistry and PCR-based detection) were used to identify HPV-positive patients, defined as those with lesions positive for both p16 and HPV-DNA [20]. Depending on tissue extent, a variable number of 1−3 × 5 μm sections was cut for each formalin-fixed paraffin-embedded (FFPE) cancer tissue. HPV-DNA detection and genotyping were achieved by PCR-based INNO-LiPA HPV Genotyping Extra II kit (Fujirebio, Malvern, PA, USA) and a TENDIGOTM instrument (Fujirebio, Malvern, PA, USA), after purifying DNA with a DNeasy Blood and Tissue Kit (Qiagen, Hilden, Germany). The expression of p16 protein was determined by a CINtec^®^ Histology Kit (Roche Diagnostics, Milan, Italy).

### 2.3. MR Imaging Protocol

MRI examinations were performed on a 1.5-T system (Optima MR 450w, GE Health-care, Milwaukee, WI, USA) with 16-channel radiofrequency coils, including a head coil, a surface neck coil, and a spine coil. The MR examination included coronal fast spin-eco (FSE) T2-weighted (T2-w) images (slice thickness, 4 mm), followed by axial FSE T2-w images (slice thickness, 3 mm), and pre-contrast axial T1-w images (slice thickness, 3 mm), acquired from the level of the skull base to the thoracic inlet. DWI were obtained via single-shot spin-echo and echo-planar imaging (slice thickness, 4 mm), with multiple b values (b = 0, 25, 50, 75, 100, 150, 300, 500, and 800 s/mm^2^). DCE-MRI was obtained through a 3D fast-spoiled gradient echo sequence (slice thickness of 6 mm, and spacing between slices 1.5 mm). In total, 30 dynamic volumes were acquired after having optimized temporal resolution (5 s) and total scanning time (5 min and 15 s). After three dynamic volumes, a contrast agent (0.1 mmol/kg bodyweight of gadopentetate dimeglumine) was injected intravenously, with a rate of 3 mL/s. According to the standard acquisition protocol, the protocol also included post-contrast T1-w images with liver acquisition with volume acceleration sequences (LAVA; slice thickness 1 mm, 214 slices) on axial and coronal planes.

### 2.4. Image Analysis

PTs and LNs were identified and manually contoured by two expert HN radiologists (F.P. and E.G.) in consensus. Anatomical T2-w images and/or post-contrast T1-w images were used for PT and LN segmentation and volume size quantification. Image visualizing and contouring were performed using 3D Slicer Software (Version 4.1.1). Based on their appearance on T2-w images and/or post-contrast T1-w images, PTs were described as having well-defined borders, as well as being exophytic, ulcerated, and necrotic, while LNs were classified as solid, cystic, or necrotic. Cystic change was defined as a homogeneous low signal intensity on T1-w images and high signal intensity on T2-w images relative to normal muscle, with the enhancement of the capsule after contrast medium with well-defined margins, homogeneous fluid content with no internal complexity, irregularity, or solid component by criteria of Goldenberg et al. [14], or with a component greater than 20% of the total volume by the criteria of Morani et al. [13]. In the case of irregular margins, it was considered a necrotic focus [13]. Radiologic extracapsular spread (rECS) of LNs has been described as irregular margins with invasion or distortion of surrounding soft tissues [13].

The diffusion parameters were derived from homemade scripts created in the MATLAB programming language (Release 2018b, The MathWorks Inc., Natick, MA, USA). To perform the data fitting, the median value of the signal within the entire lesion was calculated at each b value. Data points acquired at b values of 300, 500, and 800 s/mm^2^ were used to estimate the tissue diffusion coefficient D_t_ (in mm^2^/s) based on a mono-exponential function; ADC was similarly calculated using b values of 0, 500, and 800 s/mm^2^ [21]. The perfusion-related coefficients, *f* (fractional volume of capillary blood) and D * (perfusion-related diffusion coefficient), were not included in the analyses, considering that, in a previous investigation [15], no association was found between these variables and the HPV status.

Perfusion maps were derived from commercial software (GenIQ General, GE Advanced Workstation, Palo Alto, CA, USA). A pharmacokinetic two-compartment model was applied to DCE-MRI images to extract: K^trans^, the transfer constant between plasma and the extravascular extracellular space (EES); K_ep_, the transfer constant between EES and plasma; and v_e_, the fractional volume of EES [22]. The semi-quantitative parameter, IAUGC, which represents the initial area under the gadolinium concentration curve (calculated from the bolus arrival to the first 90 s), was also estimated. Particular attention was paid to remove arteries/veins and bones from PT/LN contours. The median of each parameter within the total lesion volume was used to perform the statistical tests.

### 2.5. Statistical Analyses

Median values and interquartile ranges were used to describe continuous variables, while frequencies and percentage values were used for categorical variables. Differences between continuous variables were assessed with the Mann–Whitney test or Student’s *t*-test, as appropriate, while the relationships between categorical variables were evaluated using the chi-square or Fisher’s Exact tests, as appropriate. The Shapiro–Wilk test was applied to evaluate the normality distribution of the data.

The chi^2^ test was used to evaluate potential differences in smoking status and alcohol intake by HPV status. Specifically, patients were defined as non-smokers if they smoked <6 packs/year, moderate smokers if they smoked 6 ≤ packs/year < 25, and strong smokers if they smoked ≥25 packs/year; patients were defined as a moderate alcoholic if they drank <1 L of wine/day and heavy alcoholic if they drank ≥1 L of wine/day.

Additional tests were conducted considering three patient subgroups according to smoking status, alcohol intake, and HPV status: specifically, HPV-negative patients, HPV-positive non-smoker (<5 pack/year), non-alcoholic patients and HPV-positive smokers (≥6 pack/year), and moderate or heavy alcoholic patients. The Kruskal–Wallis test was applied to assess the differences between the groups of patients.

A univariate logistic regression model was applied to identify variables that might play a role in the risk of HPV status in OPSCC. A multivariate logistic regression model was used with predictive variables that were significant in the univariate analyses following the forward selection method. The confidence interval for the area under the receiver operating characteristic curve (AUC) of each model was calculated with the bias-corrected and accelerated percentile bootstrap method, using 1000 replicates. The model classification ability was also evaluated in terms of accuracy, sensitivity, specificity, positive predictive value (PPV), and negative predictive value (NPV).

The statistical calculations were performed using SPSS statistical software version 21 (SPSS Inc., Chicago, IL, USA).

## 3. Results

In total, 100 patients affected by OPSCC, 72 HPV-positive and 28 HPV-negative, were prospectively included in our investigation from January 2016 to September 2019. Table 1 reports the selected patient and tumor characteristics.

Patients with HPV-related OPSCC tended to be younger and were more likely to have lower N-stage disease than patients with non-HPV-related OPSCC. The proportion of HPV-positive OPSCC was significantly affected by the tumor subsite (*p* = 0.025).

Data on smoking status were missing in one HPV-negative patient, while data on alcohol intake were missing for two HPV-negative patients and one HPV-positive patient. Regarding smoking status, 31 HPV-positive and 4 HPV-negative patients were non-smokers; 13 HPV-positive and 4 HPV-negative patients were moderate smokers; and 22 HPV-positive and 25 HPV-negative patients were strong smokers (*p* < 0.001). Regarding alcohol intake, 45 HPV-positive and 9 HPV-negative patients were non-drinkers; 19 HPV-positive and 6 HPV-negative patients were moderate alcoholics; and 4 HPV-positive and 14 HPV-negative patients were heavily alcoholic (*p* < 0.001).

No statistically significant differences in PT volumes (median value of 11.6 cm^3^ for HPV-positive group and 17.8 cm^3^ for HPV-negative group; *p* value = 0.539) and LN volumes (median value of 5 cm^3^ for HPV-positive group and 7 cm^3^ for HPV-negative group; *p* value = 0.317) were observed.

### 3.1. Multiparametric MRI Evaluation

When comparing the two patient groups, no significant differences emerged for PT characteristics (presence of well-defined borders, exophytic growth, ulceration, and necrosis) or the morphology of LNs (Table 2).

The distribution of rECS was similar in HPV-positive and HPV-negative patients, with 38 cases (60%) of ECS and 14 (54%), respectively (chi-square test, *p* = 0.107).

Conversely, ADC and D_t_ significantly differed by HPV status in PTs (*p* = 0.009 and *p* = 0.001, respectively) and LNs (*p* = 0.007 and *p* = 0.029, respectively), as shown in Table 3.

No statistically significant differences were reported in DCE perfusion parameters (K^trans^, K_ep_, v_e_, and IAUGC) between the two patient groups for both PTs and LNs (Table 4).

### 3.2. Interactions between Imaging-Based Parameters and Clinical Factors

Some interactions between imaging-based parameters, particularly ADC and D_t_, and the most relevant clinical factors associated with HPV status were evaluated. We investigated whether the tumor subsite (tonsil or base of the tongue) could significantly impact ADC and D_t_ (Supplementary Materials, Table S1). We found that the tumor subsite significantly affected ADC and D_t_ parameters of PTs (*p* = 0.001 and *p* < 0.001, respectively) but not ADC and D_t_ parameters of LNs (*p* = 0.274 and *p* = 0.243, respectively). Based on this, the differences in ADC and D_t_ of PTs by HPV status were further investigated in tonsil and base of the tongue groups, separately (Supplementary Table S2). Even though both ADC and D_t_ values of HPV-positive PTs were still lower than those of HPV-negative PTs, only in the base of the tongue was the difference in D_t_ on the significance threshold (*p* = 0.051).

Analogously, a comparison between diffusion parameters of HPV-negative, HPV-positive non-smoker, non-alcoholic patients, and HPV-negative versus HPV-positive smoker and alcoholic patients, for both PTs and LNs, was performed (Supplementary Table S3).

Lastly, we assessed whether both ADC and D_t_ could be considered independent from tumor size and rECS (Supplementary Tables S4 and S5). No (or a very weak) association was found between ADC/D_t_ and volume size, both for PTs and LNs, and ADC/Dt values did not significantly differ between LN with or without rECS. This suggested that tumor size and LN tumor invasion did not significantly impact the ADC and D_t_ parameters, independently of the HPV status of the tumor.

### 3.3. Logistic Regression Models

Univariate and multivariate logistic regression analyses for HPV positivity are reported in Table 5, including the two larger subgroups of the tumor subsite (base of the tongue, *n* = 42 and tonsil, *n* = 53). Due to the high collinearity between the ADC and D_t_ parameters, only D_t_ was included in the multivariate model.

**Figure 1:**
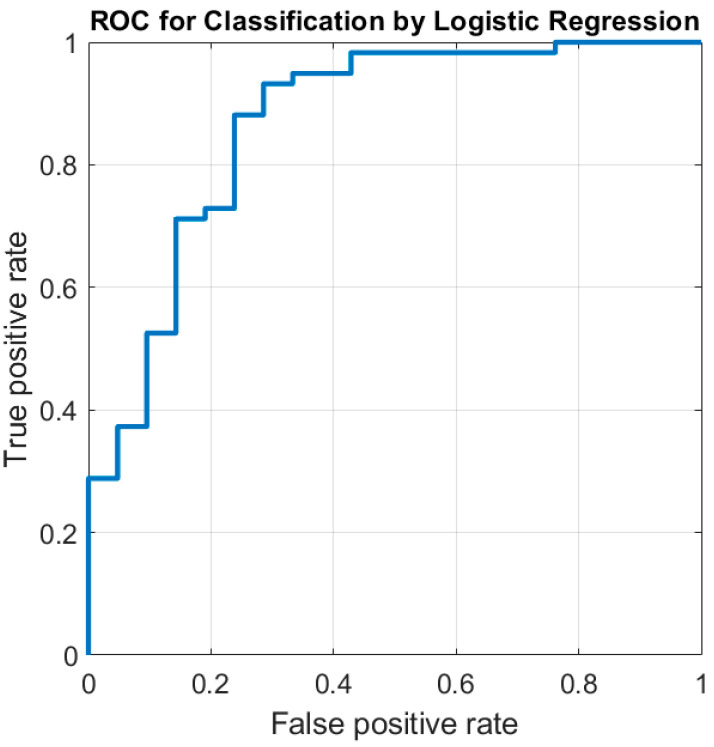
Receiver-operating characteristic (ROC) curves for predicting human papillomavirus (HPV) status of oropharyngeal squamous cell carcinoma. The AUC was 0.87 [95%CI 0.73–0.95].

Tumor subsite, alcohol intake, and D_t_ of LN were the best predictor variables to determine HPV status. The resultant accuracy was 86.3% (95%CI: 76.7–92.9%), with sensitivity of 94.9% (95%CI: 85.6–98.9%), specificity of 61.9% (95%CI: 38.4–81.9%), PPV of 87.5% (95%CI: 80.1–92.4%), and NPV of 81.3% (95%CI: 57.8–93.2%)). The ROC curve of the model is illustrated in Figure 1, the AUC of which is 0.87 (95%CI 0.73–0.95).

**Figure 2:**
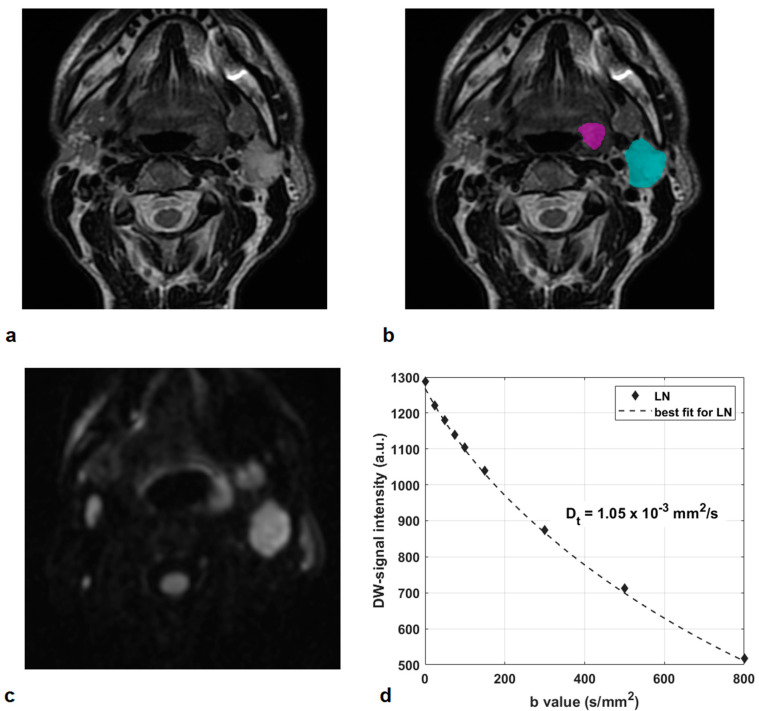
HPV-positive oropharyngeal squamous cell carcinoma of the tonsil in a 71-year-old man, non-smoker, and non-alcoholic (Patient 3): T2-w images (**a**,**b**) show the primary tumor (contour in magenta) and a large lymph node (contour in cyan). The corresponding DWI at b = 800 s/mm^2^ (**c**) and DW-signal intensity curve at increasing b values in the LN (**d**). A restricted water molecule mobility was observed in the LN, with ADC and D_t_ values of 1.17 and 1.05 × 10^−3^ mm^2^/s, respectively.

**Figure 3:**
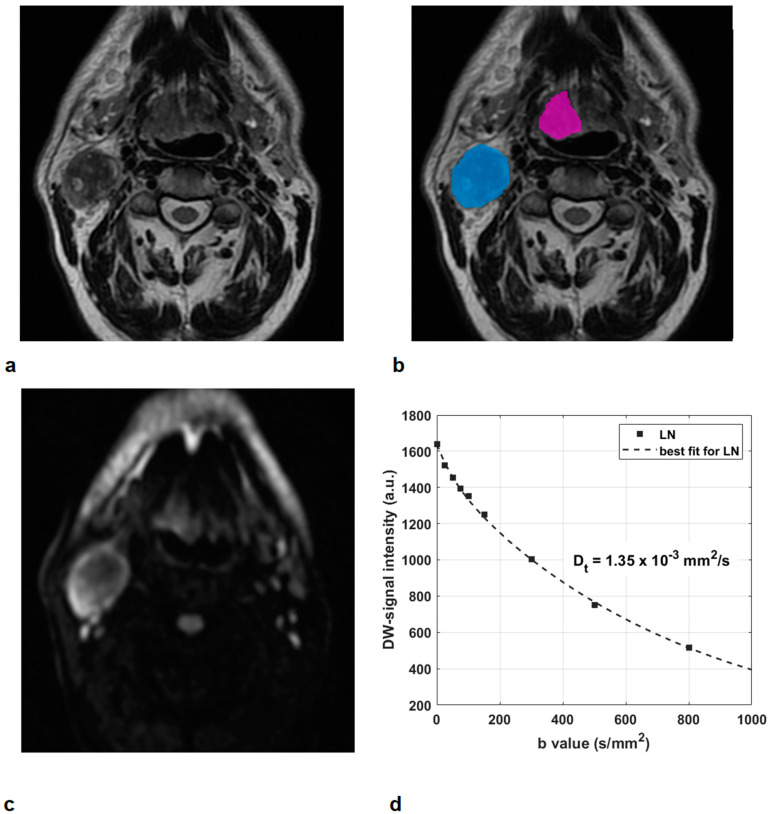
HPV-negative oropharyngeal squamous cell carcinoma of the base of the tongue in a 63-year-old moderate smoker and alcoholic man (Patient 62): T2-w images (**a**,**b**) show a primary tumor with an exophytic appearance and ulcerations (contour in magenta) and a large lymph node (LN) with extracapsular spread within IIa and IIb levels (contour in light blue). The corresponding DWI at b = 800 s/mm^2^ (**c**) and DW-signal intensity curve at increasing b values of the LN (**d**). A moderate restriction of water molecule mobility was observed in the LN, with ADC and D_t_ values of 1.52 and 1.35 × 10^−3^ mm^2^/s, respectively.

Two illustrative cases, respectively, of an HPV-positive OPSCC of the tonsil in a non-smoker and non-alcoholic man, and of an HPV-negative OPSCC of the base of the tongue in a moderate smoker and alcoholic man, are presented in Figure 2 and Figure 3.

## 4. Discussion

Recently, several investigations have focused on the dissimilar characteristics of OPSCC in relation to HPV status, using multiple techniques, i.e., CT, MRI, and PET-CT, to obtain imaging biomarkers related to lesion volume size, margins, tumor microenvironment, cellular density, vascular perfusion, and metabolism [22,23,24,25,26,27]. However, to our knowledge, this is the first study proposing a comprehensive evaluation of both anatomical and functional characteristics, based on multiparametric MRI, together with patient-related factors.

Consistent with previous studies, we reported that patients with HPV-related OPSCC had different smoking and drinking habits than HPV-negative patients [28].

Regarding morphological features in our population, we did not observe significant differences in terms of PT characteristics between the two groups; although, in HPV-positive patients, lesions with well-defined and exophytic margins were slightly more prevalent, as reported by Chan et al. [24] using both CT and MRI, and by Cantrell et al. [11] based on CT imaging.

Regarding the morphological features of OPSCC neck nodes, differences have been reported by HPV status, with the HPV-positive group showing a cystic appearance that was slightly predominant [11,13,29,30,31]. On the contrary, in our series, we found a majority of solid neck metastases for both patient groups. This aspect is important to predict the risk of treatment failure, as suggested by Rath et al. [12], who found that, in HPV-positive patients, solid neck metastases represented an independent element for treatment local failure after chemoradiation therapy together with T-stage and ECS. In another study by Barakat et al. [32], it was noticed that cystic nodes metastasis showed a shrinkage to solid ones after induction chemotherapy.

Regarding rECS, in our series, we did not find a statistically significant difference between HPV-positive and negative patients. In terms of the significance of the ECS, in patients with HPV-positive OPSCC, there are contradictory opinions: some authors [33,34] maintain that ECSs are not correlated with regional recurrence or distant metastases, while, more recently, other authors [23,35] have argued that ECSs may be associated with increased rates of locoregional recurrence and distant metastasis, with worse overall survival, and with disease-free survival. In a recent review of Park et al. [35], who evaluated the diagnostic performance of CT and MRI, sensitivity and specificity were 73% and 83% for CT and 60% and 96% for MRI, respectively; however, a lower specificity was found in patients affected by HPV-positive OPSCC (74% versus 87%), with no significant difference in sensitivity.

The recent literature demonstrates a growing interest in using multimodality techniques to obtain a quantification of functional features of tumors that are potentially associated with tissue architecture, microvascularity, or metabolic activity [29,36], intending to offer personalized treatment and management strategies [37]. Moreover, identifying HPV status from imaging biomarkers may be useful in the case of lesions hardly accessible for biopsy, as well as if a patient declines invasive procedures such as transoral robotic surgery.

The potential of DWI to discriminate OPSCC by HPV status has been already reported in earlier papers [15,24,27,36]. In this study, we used the IVIM technique to quantify both conventional ADC values and tissue diffusion coefficient D_t_ to better investigate the role of pure cellularity [38]. We found significantly lower ADC and D_t_ values of PTs in HPV-positive patients than HPV-negative patients, which is consistent with the previous literature [24,27]. However, when evaluating the potential interaction between diffusion parameters and tumor subsite, it emerged that the latter had a significant impact on ADC and D_t_ parameters of PTs, and only in the base of the tongue was the difference in D_t_ between HPV-negative and HPV-positive patients on the significance threshold.

Compared to our previous work [15], a significant difference was also reported in D_t_ and ADC for LNs in a larger population. Contrary to PTs, no differences in ADC and D_t_ parameters of LNs were found in relation to the tumor subsite, suggesting that ADC/D_t_ and tumor subsite may be considered independent predictors of HPV status; this is consistent with the results of the multivariate regression model, as discussed below.

The stronger statistical significance of D_t_, in comparison with ADC, suggest that it may be more convenient to eliminate the perfusion contribution from the diffusion coefficient estimation to more accurately investigate cellular microstructure. These differences in D_t_ were also confirmed after dividing HPV-positive patients based on smoking and alcohol habits; in fact, it can be noticed that D_t_ values of HPV-positive non-smoker non-alcoholic patients and HPV-positive smoker and alcoholic patients substantially overlap for both PTs and LNs.

It was hypothesized by Driessen et al. [39] that differences in diffusion parameters by HPV status may be connected to a smaller tumor-stromal component that is present in HPV-positive OPSCC at pathology and was correlated to ADC values, in agreement with subsequent studies [40]. In a study of Ward et al. [41], the separation in ADC estimates between HPV-negative and HPV-positive OPSCC was attributed to increased levels of lymphocytes in the latter, which may justify the decreased ADC values and the more favorable prognosis of this patient group.

The possible impact of HPV status on DCE-MRI parameters was also investigated. Consistent with earlier literature, any difference in vascularity was reported based on HPV status for primary tumors and lymph nodes [36,42,43].

The multivariate regression model had good predictive performance (AUC = 0.87) and indicated that the most significant factors associated with HPV status were tumor subsite, alcohol intake, and D_t_ of LN. In particular, tumors located in the tonsil, compared to those located in the base of the tongue, were associated with a higher risk of HPV positivity according to a recent investigation of Bos et al. [44], while moderate or heavy alcoholic patients and increased D_t_ values of lymphadenopathy were associated with a lower risk of HPV-positive OPSCC [45,46].

Some potential limitations of our study need to be acknowledged. Even though the patient population was quite large, there was a lower proportion of HPV-negative patients, which may have introduced a potential statistical sample bias. Moreover, further quantitative analyses could have been proposed, particularly on DWI, which has been demonstrated to be more informative in relation to HPV status [40]. Future investigations could also include radiomic analysis from D_t_ maps to explore the diagnostic performance of predictive multifactorial models of HPV positivity using an independent external cohort of patients to validate the models.

In conclusion, our study suggests that the combination of clinical factors, such as tumor subsite and alcohol habits, with the perfusion-free diffusion coefficient D_t_ of lymphadenopathy may help to accurately discriminate OPSCC by HPV status.

## Figures and Tables

**Table 1:** Characteristics of patients and tumors.

Patient and Tumor Characteristics		HPV-Negative	HPV-Positive	
		(*n* = 31)	(*n* = 69)	*p* Value
**Gender**		N	N	
	*Male*	27 (87%)	55 (80%)	0.543
	*Female*	4 (13%)	14 (20%)	
**Age (mean ± SD)**		67.9 ± 9.1	65.1 ± 9.5	0.158
**Tumor subsite**	*Tonsil*	11 (35%)	42 (61%)	**0.025**
	*Base of the tongue*	18 (58%)	24 (35%)	
	*Soft Palate*	1 (3%)	0	
	*Posterior Wall*	1(3%)	0	
	*Unknown*	0	3 (4%)	
T-stage *	T0	0	3(4%)	
	T1	3 (10%)	6 (9%)	
	T2	7 (23%)	23 (33%)	
	T3	3 (10%)	8 (12%)	
	T4	-	29 (42%)	
	T4a	18 (58%)	-	
N-stage *	N0	5 (16%)	6 (9%)	
	N1	3 (4%)	26 (38%)	
	N2	9 (29%)	37 (54%)	
	N3	14 (45%)	0	

* No *p* value was indicated for T and N stage categories because the disease staging based on the 8th edition of AJCC Cancer Staging Manual for HNSCC requires a priori knowledge of HPV status, and differs between HPV-positive and HPV-negative tumors. Statistically significant *p* values are **bold**.

**Table 2:** Primary tumor and nodal morphology sorted by human papillomavirus (HPV) status.

Tumor Characteristics		HPV-Negative	HPV-Positive	*p* Value
**Primary Tumors**		*n* (%)	*n* (%)	
	Well-defined borders	12 (39%)	31 (47%)	0.338
	Exophytic	7 (23%)	21 (32%)	
	Ulceration	11 (35%)	13 (20%)	
	Necrosis	1 (3%)	1 (1.5%)	
**Lymph Nodes**	Solid	13 (50%)	28 (44%)	0.495
	Cystic	2 (8%)	11 (17%)	
	Necrotic	11 (42%)	24 (38%)	

**Table 3:** ADC and D_t_ coefficients in primary tumors (PTs) and metastatic lymph nodes (LNs).

	HPV-Negative		HPV-Positive		
**PT**	*Median*	*IQR*	*Median*	*IQR*	*p Value*
*ADC*	1.451	0.388	1.255	0.378	0.006 *
D_t_	1.163	0.354	0.957	0.237	**0.001**
**LN**	*Median*	*IQR*	*Median*	*IQR*	*p Value*
*ADC*	1.333	0.468	1.090	0.286	**0.018**
D_t_	1.108	0.335	0.904	0.248	**0.005**

Abbreviations: IQR, interquartile range; ADC, apparent diffusion coefficient; D_t_, tissue diffusion coefficient. * Student’s *t*-test. Statistically significant *p* values are **bold**.

**Table 4:** Perfusion parameters from DCE-MRI in primary tumors (PTs) and metastatic lymph nodes (LNs).

	HPV-Negative		HPV-Positive		
**PT**	*Median*	*IQR*	*Median*	*IQR*	*p Value*
K^trans^ (min^−1^)	0.80	0.48	0.76	0.44	0.749
K_ep_ (min^−1^)	1.88	0.88	2.00	1.04	0.483
v_e_ (f.u)	0.38	0.21	0.41	0.15	0.654
IAUGC (a.u)	0.51	0.22	0.55	0.17	0.633
**LN**	*Median*	*IQR*	*Median*	*IQR*	*p Value*
K^trans^ (min^−1^)	0.57	0.40	0.55	0.42	0.799
K_ep_ (min^−1^)	1.84	1.34	1.60	0.92	0.642
v_e_ (f.u)	0.27	0.19	0.30	0.20	0.921
IAUGC (a.u)	0.36	0.17	0.40	0.23	0.363

Abbreviations: IQR, interquartile range; K^trans^ (min^−1^), transfer constant between plasma and EES (extravascular extracellular space); K_ep_ (min^−1^), transfer constant between EES and plasma; v_e_, fractional volume of EES; IAUGC, the initial area under gadolinium concentration curve. *p* values refer to Mann–Whitney.

**Table 5:** Uni- and multivariate logistic regression models of factors associated with HPV status.

*Selected Parameters*		*Univariate Logistic Regression Model*		*Multivariate Logistic Regression Model*	
		OR (95%CI)	*p* Value	OR (95%CI)	*p* Value
**Gender**	*Female versus Male*	3.63 (0.77–17.17)	0.103		
**Age**		0.97 (0.93–1.02)	0.262		
**Tumor subsite**	*Base of the tongue versus Tonsil*	0.35 (0.14–0.86)	**0.022**	0.09 (0.01–0.51)	**0.007**
**Smoking status**	*6–24 pack/year versus 0–5 pack/year*	0.34 (0.07–1.72)	0.191		
	*>24 pack/year versus* *0–5 pack/year*	0.12 (0.03–0.44)	**0.002**		
**Alcohol intake**	*Moderate versus No*	0.67 (0.19–2.33)	0.528	0.37 (0.05–2.63)	0.319
	*Heavy versus No*	0.05 (0.01–0.20)	**<0.001**	0.02 (0.00–0.14)	**<0.001**
**ADC of PT**		0.12 (0.02–0.58)	**0.008**		
**D_t_ of PT**		0.05 (0.01–0.43)	**0.006**		
**ADC of LN**		0.12 (0.02–0.59)	**0.009**		
**D_t_ of LN**		0.10 (0.02–0.50)	**0.005**	0.06 (0.00–0.78)	**0.032**

Statistically significant *p* values are **bold**.

## Data Availability

The data presented in this study are available in Supplementary Materials.

## Supplementary Materials

The following are available online at https://www.mdpi.com/article/10.3390/jcm10173865/s1, Table S1: ADC and D_t_ coefficients in primary tumors (PTs) and metastatic lymph nodes (LNs) by the tumor subsite; Table S2: ADC and D_t_ coefficients in primary tumors (PTs) sorted by the HPV status, in Tonsil and Base of the tongue subgroups, separately; Table S3: Comparison between imaging parameters of HPV-negative, HPV-positive non-smoker non-alcoholic patients, and HPV-positive smoker and alcoholic patients, for primary tumors (PTs) and lymph-nodes (LNs); Table S4: Results of Spearman’s correlation tests between ADC/D_t_ and volume size of primary tumor (PT) and lymph node (LN); Table S5: ADC and D_t_ coefficients in lymph nodes (LNs) with and without extra-capsular spread (ECS).

## References

[1] Bennardo L., Bennardo F., Giudice A., Passante M., Dastoli S., Morrone P., Provenzano E., Patruno C., Nisticò S.P. (2021). Local chemotherapy as an adjuvant treatment in unresectable squamous cell carcinoma: What do we know so far?. Curr. Oncol..

[2] Pentangelo G., Nisticò S.P., Provenzano E., Cisale G.Y., Bennardo L.G. (2021). Topical 5% imiquimod sequential to surgery for hpv-related squamous cell carcinoma of the lip. Medicina.

[3] Marur S., D’Souza G., Westra W.H., Forastiere A.A. (2010). HPV-associated head and neck cancer: A virus-related cancer epidemic. Lancet Oncol..

[4] Li H., Torabi S.J., Yarbrough W.G., Mehra S., Osborn H.A., Judson B. (2018). Association of human papillomavirus status at head and neck carcinoma subsites with overall survival. JAMA Otolaryngol. Head Neck Surg..

[5] Lewis J.S., Thorstad W.L., Chernock R.D., Haughey B.H., Yip J.H., Zhang Q., El-Mofty S.K. (2010). p16 positive oropharyngeal squamous cell carcinoma: An entity with a favorable prognosis regardless of tumour HPV status. Am. J. Surg. Pathol..

[6] Ragin C.C., Taioli E. (2007). Survival of squamous cell carcinoma of the head and neck in relation to human papillomavirus infection: Review and meta-analysis. Int. J. Cancer.

[7] Sivars L., Landin D., Haeggblom L., Tertipis N., Grün N., Bersani C., Marklund L., Ghaderi M., Näsman A., Ramqvist T. (2016). Diagnostic role of detecting HPV in a FNAC of metastatic laterocervical lymph node in a case of occult HPV-related head and neck squamous cell carcinoma. Pathologica.

[8] Paver E.C., Currie A., Gupta R., Dahlstrom J.E. (2020). Human papilloma virus related squamous cell carcinomas of the head and neck: Diagnosis, clinical implications and detection of HPV. Pathology.

[9] Takesa R.P., Kaandersb J.H.A.M., van Herpenc C.M.L., Merkxd M.A.W., Slootwege P.J., Melchers W.J.G. (2016). Human papillomavirus detection in fine needle aspiration cytology of lymph node metastasis of head and neck squamous cell cancer. J. Clin. Virol..

[10] Fujita A., Buch K., Li B., Kawashima Y., Qureshi M.M., Sakai O. (2016). Difference between HPV-positive and HPV-negative non-oropharyngeal head and neck cancer: Texture analysis features on CT. J. Comput. Assist. Tomogr..

[11] Cantrell S.C., Peck B.W., Li G., Wei Q., Sturgis E.M., Ginsberg L.E. (2013). Differences in imaging characteristics of HPV-positive and HPV-Negative oropharyngeal cancers: A blinded matched-pair analysis. AJNR Am. J. Neuroradiol..

[12] Rath T.J., Narayanan S., Hughes M.A., Ferris R.L., Chiosea S.I., Branstetter B.F. (2017). Solid lymphnodes as an imaging biomarker for risk stratification in human papillomavirus-related oropharyngeal squamous cell carcinoma. AJNR Am. J. Neuroradiol..

[13] Morani A.C., Eisbruch A., Carey T.E., Hauff S.J., Walline H.M., Mukherji S.K. (2013). Intranodal cystic changes: A potential radiologic signature/biomarker to assess the human papillomavirus status of cases with oropharyngeal malignancies. J. Comput. Assist. Tomogr..

[14] Goldenberg D., Begum S., Westra W.H., Khan Z., Sciubba J., Pai S.I., Califano J.A., Tufano R.P., Koch W.M. (2008). Cystic lymph node metastasis in patients with head and neck cancer: An HPV-associated phenomenon. Head Neck.

[15] Vidiri A., Marzi S., Gangemi E., Benevolo M., Rollo F., Farneti A., Marucci L., Spasiano F., Sperati F., Di Giuliano F. (2019). Intravoxel incoherent motion diffusion-weighted imaging for oropharyngeal squamous cell carcinoma: Correlation with human papillomavirus Status. Eur. J. Radiol..

[16] Surov A., Meyer H.J., Winter K., Richter C., Hoehn A.K. (2018). Histogram analysis parameters of apparent diffusion coefficient reflect tumor cellularity and proliferation activity in head and neck squamous cell carcinoma. Oncotarget.

[17] Surov A., Meyer H.J., Gawlitza M., Hohn A.K., Boehm A., Kahn T., Stumpp P. (2017). Correlations between DCE MRI and histopathological parameters in head and neck squamous cell carcinoma. Transl. Oncol..

[18] Unetsubo T., Konouchi H., Yanagi Y., Murakami J., Fujii M., Matsuzaki H., Hisatomi M., Nagatsuka H., Asaumi J. (2009). Dynamic contrast-enhanced magnetic resonance imaging for estimating tumour proliferation and microvessel density of oral squamous cell carcinomas. Oral Oncol..

[19] O’Sullivan B., Lydiatt W.M., Haughey B.H., Brandwein-Gensler M., Glastonbury C.M., Shah J.P., Amin M.B., Edge S.B., Greene F.L. (2017). HPV-mediated (p16+) oropharyngeal cancer. AJCC Cancer Staging Manual.

[20] Mena M., Taberna M., Tous S., Marquez S., Clavero O., Quiros B., Lloveras B., Alejo M., Leon X., Quer M. (2018). Double positivity for HPV-DNA/p16ink4a is the biomarker with strongest diagnostic accuracy and prognostic value for human papillomavirus related oropharyngeal cancer patients. Oral Oncol..

[21] Taouli B., Beer A.J., Chenevert T., Collins D., Lehman C., Matos C., Padhani A.R., Rosenkrantz A.B., Shukla-Dave A., Sigmund E. (2016). Diffusion-weighted imaging outside the brain: Consensus statement from an ISMRM-sponsored workshop. J. Magn. Reson. Imaging.

[22] Tofts P.S., Brix G., Buckley D.L., Evelhoch L., Henderson E., Knopp M.V., Larsson H.B., Lee T.Y., Mayr N.A., Parker G.J. (1999). Estimating kinetic parameters from dynamic contrast enhanced T1- weighted MRI of a diffusable tracer: Standardised quantities and symbols. J. Magn. Reson. Imaging.

[23] Huang S.H., O’Sullivan B., Su J., Bartlett E., Kim J., Waldron J.N., Ringash J., de Almeida J.R., Bratman S., Hansen A. (2020). Prognostic importance of radiologic extranodal extension in HPV-positive oropharyngeal carcinoma and its potential role in refining TNM-8 cN-classification. Radiother. Oncol..

[24] Chan M.W., Higgins K., Enepekides D., Poon I., Symons S.P., Moineddin R., Weinreb I., Shearkhani O., Chen A., Beelen J. (2016). Radiologic differences between human papillomavirus-related and human papillomavirus-unrelated oropharyngeal carcinoma on diffusion-weighted imaging. J. Otorhinolaryngol. Relat. Spec..

[25] Bisdas S., Seitz O., Middendorp M., Chambron-Pinho N., Bisdas T., Vogl T.J., Hammerstingl R., Ernemann U., Mack M.G. (2010). An exploratory pilot study into the association between microcirculatory parameters derived by MRI-based pharmacokinetic analysis and glucose utilisation estimated by PET-CT imaging in head and neck cancer. Eur. Radiol..

[26] Jansen J.F., Schoder H., Lee N.Y., Wang Y., Pfister D.G., Fury M.G., Stambuk H.E., Humm J.L., Koutcher J.A., Shukla-Dave A. (2010). Noninvasive assessment of tumour microenvironment using dynamic contrast-enhanced magnetic resonance imaging and 18F-fluoromisonidazole positron emission tomography imaging in neck nodal metastases. Int. J. Radiat. Oncol. Biol. Phys..

[27] Nakahira M., Saito N., Yamaguchi H., Kuba K., Sugasawa M. (2014). Use of quantitative diffusion-weighted magnetic resonance imaging to predict human papilloma virus status in patients with oropharyngeal squamous cell carcinoma. Eur. Arch. Otorhinolaryngol..

[28] Huang S.H., O’Sullivan B., Waldron J. (2018). The current state of biological and clinical implications of human papillomavirus-related oropharyngeal cancer. Semin. Radiat. Oncol..

[29] Kawaguchi M., Kato H., Tomita H., Hara A., Suzui N., Miyazaki T., Matsuo M. (2020). Comparison of imaging findings between human papillomavirus-positive and -negative squamous cell carcinomas of the maxillary sinus. J. Clin. Imaging Sci..

[30] Kaka A.S., Kumar B., Kumar P., Wakely P.E., Kirsch C.M., Old M.O., Ozer E., Agrawal A., Carrau R.E., Schuller D.E. (2013). Highly aggressive human papillomavirus-related oropharyngeal cancer: Clinical, radiologic, and pathologic characteristics. Oral Surg. Oral Med. Oral Pathol. Oral Radiol..

[31] Jo Y.H., Jung C.K., Sun D.I., Park J.O., Cho K.J., Kim M.S. (2012). High-risk human papillomavirus and cervical lymph node metastasis in patients with oropharyngeal cancer. Head Neck.

[32] Barakat E., Ginat D.T. (2020). Differential change in size of human papillomavirus-positive cystic versus solid squamous cell carcinoma lymph node metastases in response to induction chemotherapy. J. Comput. Assist. Tomogr..

[33] Sinha P., Lewis J.S., Piccirillo J.F., Kallogjeri D., Haughey B.H. (2012). Extracapsular spread and adjuvant therapy in human papillomavirus-related, p16-positive oropharyngeal carcinoma. Cancer.

[34] Lewis J.S., Carpenter D.H., Thorstad W.L., Zhang Q., Haughey B.H. (2011). Extracapsular extension is a poor predictor of disease recurrence in surgically treated oropharyngeal squamous cell carcinoma. Mod. Pathol..

[35] Park S.I., Guenette J.P., Suh C.H., Hanna G.J., Chung S.R., Baek J.H., Lee J.H., Choi Y.J. (2020). The diagnostic performance of CT and MRI for detecting extranodal extension in patients with head and neck squamous cell carcinoma: A systematic review and diagnostic meta-analysis. Eur. Radiol..

[36] Han M., Leeb S.J., Leec D., Kima S.Y., Choi J.W. (2018). Correlation of human papilloma virus status with quantitative perfusion/diffusion/metabolic imaging parameters in the oral cavity and oropharyngeal squamous cell carcinoma: Comparison of primary tumour sites and metastatic lymph nodes. Clin. Radiol..

[37] Caudell J., Torres-Roca J.F., Gillies R.J., Enderling H., Kim S., Rishi A., Moros E.G., Harrison L.B. (2017). The future of personalised radiotherapy for head and neck cancer. Lancet Oncol..

[38] Le Bihan D., Turner R. (1992). The capillary network: A link between IVIM and classical perfusion. Magn. Reson. Med..

[39] Driessen J.P., Van Bemmel A.J., van Kempen P.M., Janssen L.M., Terhaard C.H.J., Pameijer F.A., Willems S.M., Stegeman I., Grolman W., Philippens M.E.P. (2016). Correlation of human papillomavirus status with apparent diffusion coefficient of diffusion-weighted MRI in head and neck squamous cell carcinomas. Head Neck.

[40] de Perrot T., Lenoir V., Domingo Ayllón M., Dulguerov N., Pusztaszeri M., Becker M. (2017). Apparent diffusion coefficient histograms of human papillomavirus-positive and human papillomavirus-negative head and neck squamous cell carcinoma: Assessment of tumour heterogeneity and comparison with histopathology. AJNR Am. J. Neuroradiol..

[41] Ward M.J., Thirdborough S.M., Mellows T., Riley C., Harris S., Suchak K., Webb A., Hampton C., Patel N.N., Randall C.J. (2014). Tumour-infiltrating lymphocytes predict for outcome in HPV-positive oropharyngeal cancer. Br. J. Cancer.

[42] Vidiri A., Gangemi E., Ruberto E., Pasqualoni R., Sciuto R., Sanguineti G., Farneti A., Benevolo M., Rollo F., Sperati F. (2020). Correlation between histogram-based DCEMRI parameters and 18F-FDG PET values in oropharyngeal squamous cell carcinoma: Evaluation in primary tumours and metastatic nodes. PLoS ONE.

[43] Jansen J.F., Schoder H., Lee N.Y., Stambuk H.E., Wang Y., Fury M.G., Patel S.G., Pfister D.G., Shah J.P., Koutcher J.A. (2012). Tumour metabolism and perfusion in head and neck squamous cell carcinoma: Pre-treatment multimodality imaging with 1H magnetic resonance spectroscopy, dynamic contrast-enhanced MRI, and [18F] FDG-PET. Int. J. Radiat. Oncol. Biol. Phys..

[44] Bos P., van den Brekel M.W.M., Gouw Z.A.R., Al-Mamgani A., Waktola S., Aerts H.J.W.L., Beets-Tan R.G.H., Castelijns J.A., Jasperse B. (2021). Clinical variables and magnetic resonance imaging-based radiomics predict human papillomavirus status of oropharyngeal cancer. Head Neck.

[45] Kawakita D., Matsuo K. (2017). Alcohol and head and neck cancer. Cancer Metastasis Rev..

[46] Payabvash S., Chan A., Jabehdar Maralani P., Malhotra A. (2019). Quantitative diffusion magnetic resonance imaging for prediction of human papillomavirus status in head and neck squamous-cell carcinoma: A systematic review and meta-analysis. Neuroradiol. J..

