# Supplementary material for: Multiparametric MRI Evaluation of Oropharyngeal Squamous Cell Carcinoma. A Mono-Institutional Study
Source: J Clin Med. 2021 Aug 28;10(17):3865. doi: 10.3390/jcm10173865 (PMC8432241; doi:10.3390/jcm10173865)
Supplement: Supplementary file 1 [file jcm-10-03865-s001.zip › jcm-1320981-SI/jcm-1320981-supplementary-final/SupplementaryTables_proof.pdf]

Supplementary Tables

Supplementary Table S1 ADC and D_t coefficients in primary tumors (PTs) and metastatic lymph nodes (LNs) by the tumor subsite.

	Tonsil		Base of the tongue		Mann-Whitney test
**PT**	*Median*	*IQR*	*Median*	*IQR*	*P value*
ADC	1.19	1.04-1.39	1.44	1.28-1.61	**0.001**
D_t	0.92	0.78-1.04	1.13	0.99-1.26	**<0.001**
**LN**	*Median*	*IQR*	*Median*	*IQR*	*P value*
ADC	1.12	0.94-1.32	1.18	1.03-1.35	0.274
D_t	0.92	0.79-1.09	0.99	0.82-1.22	0.243

Supplementary Table S2 ADC and D_t coefficients in primary tumors (PTs) sorted by the HPV status, in Tonsil and Base of the tongue subgroups, separately.

		HPV-negative		HPV-positive		Mann-Whitney test
**Tonsil**	**PT**	*Median*	*IQR*	*Median*	*IQR*	*P value*
	ADC	1.33	0.95-2.02	1.16	1.03-1.33	0.185
	D_t	1.06	0.79-1.36	0.90	0.78-1.01	0.163*
**Base of the tongue**	**PT**	*Median*	*IQR*	*Median*	*IQR*	*P value*
	ADC	1.46	1.32-1.64	1.42	1.19-1.61	0.346*
	D_t	1.18	1.03-1.36	1.03	0.91-1.23	**0.051**

*Student T test

Supplementary Table S3 Comparison between imaging parameters of HPV-negative, HPV-positive non-smoker non-alcoholic patients, and HPV-positive smoker and alcoholic patients, for primary tumors (PTs) and lymph-nodes (LNs)

**Parameter**	***HPV-negative***	***HPV-positive nonsmokers non-alcoholic***	***HPV-positive smokers alcoholic***	*P value*
PT	**(*n*=28)**	**(*n*=24)**	**(*n*=38)**	
**ADC(10^{-3} mm^2/s)**	1.45 (1.32-1.6)	1.33 (1.15-1.45)	1.20 (1.07-1.34)	**0.016**
**D_t(10^{-3} mm^2/s)**	1.16 (1.03-1.3)	0.96 (0.87-1.03)	0.93 (0.84-1.04)	**0.005**
LN	**(*n*=25)**	**(*n*=23)**	**(*n*=40)**	
**ADC(10^{-3} mm^2/s)**	1.33 (1.02-1.48)	1.13 (1.03-1.25)	1.08 (0.93-1.23)	0.055
**D_t(10^{-3} mm^2/s)**	1.11 (0.96-1.30)	0.93 (0.79-1.08)	0.90 (0.78-0.99)	**0.018**

Median values (95% confidence interval). P values refer to Kruskall-Wallis test. Statistically significant p-values are **bold**. Abbreviations: ADC, apparent diffusion coefficient; D_t, tissue diffusion coefficient.

Supplementary Table S4 Results of Spearman's correlation tests between ADC/D_t and volume size of primary tumor (PT) and lymph node (LN)

	ADC		***D_t***	
	Spearman's Rho (95%CI)	*p-value*	*Spearman's Rho(95%CI)*	*p-value*
PT volume	-0.005 (-0.212,0.202)	0.963	0.111 (-0.100,0.312)	0.298
LN volume	0.189 (-0.022,0.383)	0.078	0.203 (-0.007,0.395)	0.059

Supplementary Table S5 ADC and D_t coefficients in lymph nodes (LNs) with and without extra-capsular spread (ECS)

	**with ECS**		**no ECS**		
**LN**	*Median*	*95%CI*	*Median*	*95%CI*	*P value*
ADC	1.17	(1.07,1.25)	1.09	(1.02,1.21)	0.333
D_t	0.95	(0.88,1.06)	0.90	(0.80,1.05)	0.168

Abbreviation: 95%CI, 95% confidence interval. P values refer to Mann-Whitney test.
